# Prevalence of *Cryptosporidium parvum*/*hominis*, *Entamoeba histolytica* and *Giardia lamblia* among Young Children with and without Diarrhea in Dar es Salaam, Tanzania

**DOI:** 10.1371/journal.pntd.0004125

**Published:** 2015-10-09

**Authors:** Marit G. Tellevik, Sabrina J. Moyo, Bjørn Blomberg, Torunn Hjøllo, Samuel Y. Maselle, Nina Langeland, Kurt Hanevik

**Affiliations:** 1 National Centre for Tropical Infectious Diseases, Department of Medicine, Haukeland University Hospital, Bergen, Norway; 2 Department of Clinical Science, University of Bergen, Bergen, Norway; 3 Department of Microbiology and Immunology, Muhimbili University of Health and Allied Sciences, Dar es Salaam, Tanzania; 4 Department of Medicine, Haukeland University Hospital, Bergen, Norway; University of Melbourne, AUSTRALIA

## Abstract

**Background:**

Although enteroparasites are common causes of diarrheal illness, few studies have been performed among children in Tanzania. This study aimed to investigate the prevalence of *Cryptosporidium parvum*/*hominis*, *Entamoeba histolytica* and *Giardia lamblia* among young children in Dar es Salaam, Tanzania, and identify risk factors for infection.

**Methodology/Principal Findings:**

We performed an unmatched case-control study among children < 2 years of age in Dar es Salaam, recruited from August 2010 to July 2011. Detection and identification of protozoans were done by PCR techniques on DNA from stool specimens from 701 cases of children admitted due to diarrhea at the three study hospitals, and 558 controls of children with no history of diarrhea during the last month prior to enrollment. The prevalence of *C*. *parvum*/*hominis* was 10.4% (84.7% *C*. *hominis*), and that of *G*. *lamblia* 4.6%. *E*. *histolytica* was not detected. The prevalence of *Cryptosporidium* was significantly higher in cases (16.3%) than in controls (3.1%; P < 0.001; OR = 6.2; 95% CI: 3.7–10.4). *G*. *lamblia* was significantly more prevalent in controls (6.1%) than in cases (3.4%; P = 0.027; OR = 1.8; 95% CI: 1.1–3.1). *Cryptosporidium* infection was found more often in HIV-positive (24.2%) than in HIV-negative children (3.9%; P < 0.001; OR = 7.9; 95% CI: 3.1–20.5), and was also associated with rainfall (P < 0.001; OR = 2.41; 95% CI: 1.5–3.8). Among cases, stunted children had significantly higher risk of being infected with *Cryptosporidium* (P = 0.011; OR = 2.12; 95% CI: 1.2–3.8). *G*. *lamblia* infection was more prevalent in the cool season (P = 0.004; OR = 2.2; 95% CI: 1.3–3.8), and more frequent among cases aged > 12 months (P = 0.003; OR = 3.5; 95% CI: 1.5–7.8). Among children aged 7–12 months, those who were breastfed had lower prevalence of *G*. *lamblia* infection than those who had been weaned (P = 0.012).

**Conclusions:**

*Cryptosporidium* infection is common among young Tanzanian children with diarrhea, particularly those living with HIV, and infection is more frequent during the rainy season. *G*. *lamblia* is frequently implicated in asymptomatic infections, but rarely causes overt diarrheal illness, and its prevalence increases with age.

## Author Summary

Diarrheal diseases are a leading cause of disease and deaths among young children. In Africa they contribute to more than one tenth of childhood deaths. Parasites like *Cryptosporidium*, *Entamoeba histolytica* and *Giardia lamblia* are all common causes of diarrheal illness, but there are few studies on these enteroparasites among Tanzanian children. In this case-control study, we included 701 cases and 558 controls, all < 2 years of age, in Dar es Salaam, Tanzania. We assessed the prevalence of *C*. *parvum*/*hominis*, *E*. *histolytica* and *G*. *lamblia* by PCR, and the association with potential risk factors such as demographic data, clinical symptoms, HIV status and seasonality. One or more parasites were found in 14.9% of the samples. *C*. *parvum*/ *hominis* and *G*. *lamblia* were found in 10.4% and 4.6%, respectively, while *E*. *histolytica* was not found in any of the samples. The prevalence of *Cryptosporidium* was high, particularly in children with HIV, and its prevalence increased during the rainy season. Among cases, *Cryptosporidium* was found more frequently in stunted children, although any causal association could not be established in the current study. *G*. *lamblia* was more often implicated in asymptomatic infections than in overt diarrheal illness. The prevalence of *G*. *lamblia* increased with age, and breastfeeding seemed to protect the children from *G*. *lamblia*. This study presents relevant information about the prevalence and clinical characteristics of these intestinal parasites in Tanzanian children.

## Introduction

Diarrheal disease is a leading cause of mortality and morbidity in young children, estimated to cause more than 760 000 annual deaths among children < 5 years of age [1], with 72% of these deaths occurring in children < 2 years of age [2]. Globally, diarrheal diseases take more lives than malaria and HIV together [3]. While malaria is the leading cause of child deaths in the African region, diarrheal diseases still contribute to more than one tenth of deaths in African children [3], and sub-Saharan Africa accounts for half of all global childhood deaths from diarrheal diseases [2]. Diarrheal diseases can be caused by various bacteria, viruses and parasites. Among the main infectious diarrheagenic pathogens, *Cryptosporidium* spp. results in the most deaths among children < 5 years of age [4]. Two other enteric protozoan parasites, *Giardia lamblia* (synonymous with *G*. *intestinalis*, *G*. *duodenalis*) and *Entamoeba histolytica* also contribute, but to a lesser extent [5]. The genus *Cryptosporidium* consists of approximately 20 different species, with *C*. *hominis* and *C*. *parvum* being the major species infecting humans. Transmission occurs via the fecal-oral route from human and animal reservoirs. In immunocompetent hosts, cryptosporidiosis is usually self-limiting, but in developing countries it contributes to persistent diarrhea in children and is a major enteric pathogen causing chronic diarrhea in HIV-positive patients [6]. *G*. *lamblia* is a known cause of diarrheal disease world-wide, but is more frequently encountered in developing countries [6]. It causes the diarrheal illness giardiasis, but can also be asymptomatic [7]. *E*. *histolytica* causes amoebiasis, with a wide spectrum of clinical presentations, ranging from asymptomatic infection to diarrhea, amoebic colitis, amoebic dysentery and abscesses in the liver, lungs or brain. It is endemic in several parts of the world. However, while symptomatic disease is rare, the outcome is often severe [8, 9]. All these three parasites can cause waterborne outbreaks, and also foodborne outbreaks have been reported [6,10].

Although varying in designs and settings, other studies from sub-Saharan Africa have found prevalence ranging up to 30.5% for *Cryptosporidium* spp. [11], 10.7% for *E*. *histolytica/ dispar* [11] and 60.1% for *G*. *lamblia* [12] in children < 5 years of age with diarrhea. Few studies of enteroparasites among young children with diarrheal illness have been performed in Tanzania [13–17], and most of these had limited study populations. Seasonal differences for several pathogens causing diarrheal disease has been reported [11,13,18].

The objectives of the present study were to investigate the prevalence of *C*. *parvum*/ *hominis*, *E*. *histolytica* and *G*. *lamblia* among young children in Dar es Salaam, Tanzania, and to identify risk factors for infection. The results of this study may contribute useful information about prevalence and risk factors for these intestinal parasites in Tanzania.

## Methods

### Ethics statement

The study was approved by the Senate Research and Publication Committee of Muhimbili University of Health and Allied Sciences in Dar es Salaam, Tanzania, by the Regional Committee for Medical and Health Research Ethics (REK) in Norway, and by the respective hospital authorities at the three study hospitals. Written informed consent was obtained from the parents or guardian on behalf of all the children enrolled in the study.

### Study population

The study population and data collection have previously been described [19]. Briefly, this prospective study was performed between August 2010 and July 2011, in Dar es Salaam, Tanzania, covering both the dry and the wet seasons. A total of 1266 children < 2 years of age were recruited. Diarrhea was defined as three or more watery stools within 24 hours. An episode of diarrhea was considered over when two consecutive days pass without diarrhea. An episode of acute diarrhea was defined as duration between 24 hours and less than 14 days. Persistent diarrhea was defined as diarrhea for 14 days or more. Cases (N = 705) were children admitted due to diarrhea at one of the three major hospitals in Dar es Salaam; Muhimbili National Hospital, Amana and Temeke Municipal district hospitals. Controls (N = 561) were children with no history of diarrhea during the last month prior to enrollment. A standardized questionnaire and patient files were used for collection of demographic and clinical information. Weight for age (WAZ), length for age (LAZ) and weight for length (WLZ) Z-scores were calculated using EPI Info (USD, Inc., Stone Mountain, GA). Children were categorized to have normal nutritional status, mild or severe malnutrition using Z-scores according to WHO criteria.

Meteorological data for the region of Dar es Salaam for each month of the study period were collected from Global Historical Climatology Network (GHCND) Monthly Summaries database, available at http://www.ncdc.noaa.gov. The rainy season was defined as the months with the heaviest rainfall in mm precipitation; October—December and March—May. The dry season was defined as the months with least rainfall in mm precipitation; August–September, January–February and June–July. The hot season was defined as the months with the highest mean temperature; October–March. The cool season was defined as the months with the lowest mean temperature; August—September and April–July.

### Sample material

One stool specimen from each child was collected on inclusion in the study, and frozen at -70°C on the day of collection.

### Multiplex real-time PCR for detection of protozoans

For extraction of DNA, 50 mg of the stool sample was mixed 1:10 with Bacterial Lysis Buffer (Roche Diagnostics, Mannheim, Germany), and centrifuged at 13 000 x g for 3 min. DNA was extracted from 200 μl supernatant using the Magna Pure LC High Performance Total Nucleic Acid Isolation Kit (Roche Applied Science, Mannheim, Germany). DNA was eluted and stored at—70°C until PCR analysis. A multiplex real-time PCR for *C*. *parvum*/ *hominis*, *E*. *histolytica* and *G*. *lamblia*, using Phocid Herpes Virus 1 (PhHV–1) as an internal control, was performed with previously published primers and probes, with some changes for the labelling. All oligonucleotides used are listed in Table 1. They were purchased from Applied Biosystems, Cheshire, UK (primers and probes for *C*. *parvum*/ *hominis*, *E*. *histolytica*), and from TIB MOLBIOL, Berlin, Germany (primers and probes for *G*. *lamblia* and PhHV–1). Each PCR test was performed in a 25 μl reaction mixture. The reaction mixture included: 1 x HotStarTaq Plus Master Mix (Qiagen, Hilden, Germany), 0.5 μg/ μl BSA (New England Biolabs, Inc., Ipswich, MA) additional 3.5 mM of MgCl_2_, concentrations of primers and probes as previously published [20], and water. One μl of PhHV–1 (diluted to give a Cq-value of approximately 32) and 4 μl of DNA sample were added to the reaction mixture. The fourplex real-time PCR assay was performed using a LightCycler 480 Instrument II (Roche Diagnostics), with cycling conditions as follows: 95°C for 5 min, followed by 45 cycles at 95°C for 15 s, 60°C for 30 s and 72°C for 30 s each, and then cooled to 40°C for 30 s. All samples were run on LightCycler 480 Multiwell Plate 96, white (Roche), and sealed with LightCycler 480 Sealing Foil (Roche). Each run included duplicate of a positive mixed control and multiple no-template controls. Dilution series, five-fold or ten-fold, of DNA extracted from each of the four pathogens were used to make a standard curve to determine the efficiency of the PCR. The PCR was repeated for samples with weak positive or uncertain results. A unidirectional workflow pre- to post-PCR was enforced, and preparation of PCR reaction mixture, DNA preparations and PCR were carried out in facilities physically separate from each other.

**Table 1:** Primers and probes used in this study.

Target organism and oligo^1^	Oligonucleotide sequences (5’– 3’)	Reference
*Cryptosporidium parvum/ Cryptosporidium hominis*		
Sense primer	CTT TTT ACC AAT CAC AGA ATC ATC AGA	[20]
Antisense primer	TGT GTT TGC CAA TGC ATA TGA A	
Probe^2^	FAM-TCG ACT GGT ATC CCT ATA A-MGBNFQ	
*Giardia lamblia* ^*3*^		
Giardia-80F	GAC GGC TCA GGA CAA CGG TT	[24]
Giardia-127R	TTG CCA GCG GTG TCC G	
Giardia-105Tlc^2^	LC610-CCC GCG GCG GTC CCT GCT AG-BBQ	
*Entamoeba histolytica*		
Ehd-239F	ATT GTC GTG GCA TCC TAA CTC A	[24]
Ehd-88R	GCG GAC GGC TCA TTA TAA CA	
Histolytica-96T	VIC-TCA TTG AAT GAA TTG GCC ATT T-MGBNFQ	
PhHV		
PhHV-267s	GGG CGA ATC ACA GAT TGA ATC	[47]
PhHV-337-as	GCG GTT CCA AAC GTA CCA A	
PhHV-305tq^2^	LC670-TTT TTA TGT GTC CGC CAC CAT CTG GAT C-BBQ	
*Cryptosporidium* SSU rRNA		
CRU18SF	GAG GTA GTG ACA AGA AAT AAC AAT ACA GG	[21]
CRU18SR	CTG CTT TAA GCA CTC TAA TTT TCT CAA AG	[21]
CRU18STM	FAM-TAC GAG+ CTT TTT AA+C TG+C AAC AA- BHQ1	[22]
*Cryptosporidium parvum*		
CRULib13F	TCC TTG AAA TGA ATA TTT GTG ACT CG	[21]
CRULib13RCp	TTA ATG TGG TAG TTG CGG TTG AAC	[21]
CRULib13TMCp^2^	HEX-TAT CT+C TT+C GTA G+CG GCG TA-BHQ1	[22]
*Cryptosporidium hominis*		
CRULib13F	See above	[21]
CRULib13RCh	AAA TGT GGT AGT TGC GGT TGA AA	[21]
CRULib13TMCh^2^	ROX-CTT A+CT T+CG TGG+ CGG CGT-BHQ2	[22]
*Giardia lamblia* ^*4*^		
AL3543	AAATIATGCCTGCTCGTCG	[23]
AL3546	CAAACCTTITCCGCAAACC	
AL3544	CCCTTCATCGGIGGTAACTT	
AL3545	GTGGCCACCACICCCGTGCC	

^1^Oligo, Name of the oligonucleotide

^2^Probe labelled with different fluorophore than used in the reference

^3^Primers and probe for detection of *Giardia lamblia*

^4^Primers for typing of *Giardia lamblia*

### Identification of *Cryptosporidium* species

To identify the *Cryptosporidium* isolates as *C*. *parvum* or *C*. *hominis*, primers and probes as described by Hadfield *et al*. and Lange *et al*., and purchased from TIB MOLBIOL, were applied [21,22]. LNA converted probes were used and some changes performed for the labelling, see Table 1. Each reaction contained 1x of LightCycler FastStart DNA Master HybProbe (Roche), 0.5 μM of each of the five primers, 0.2 μM of each of the three probes, additional 1.5 mM of MgCl_2_, 5 μl of template and water adjusted to a total volume of 20 μl. The triplex real-time PCR assay was performed using the LightCycler 480 Instrument II (Roche Diagnostics), with cycling conditions as follows: 95°C for 10 min, followed by 55 cycles at 95°C for 15 s, 60°C for 30 s and 72°C for 1 min each, and then cooled to 40°C for 30 s. All samples were run on LightCycler 480 Multiwell Plate 96, white (Roche) and sealed with LightCycler 480 Sealing Foil (Roche). Each run included positive controls and multiple no-template controls. For samples that were negative, had an uncertain or very weak positive result, the PCR was repeated without the genus-specific (SSU rRNA) primers and probe.

### Genotyping of *G*. *lamblia*

To identify the *G*. *lamblia* isolates as assemblages A or B, a nested-PCR method targeting the triosephosphate isomerase (TPI) gene as described by Sulaiman et al., but with doubled concentration of MgCl_2_, was used [23]. PCR cycling conditions for the first PCR were 95°C for 5 min, followed by 35 cycles at 95°C for 45 s, 50°C for 45 s and 72°C for 1 min each, and a final extension at 72°C for 7 min. For the second PCR, cycling conditions were 95°C for 5 min, followed by 40 cycles at 95°C for 30 s, 59°C for 30 s and 72°C for 15 s each, and a final extension at 72°C for 1 min. Primers were purchased from TIB MOLBIOL. PCR products were analyzed by gel electrophoresis and both strands sequenced using BigDye Terminator v1.1 Cycle Sequencing Kit (Applied Biosystems, Foster City, CA, USA) and an ABI PRISM 3730 DNA Analyzer (Applied Biosystems). A consensus sequence was created for each PCR-product and aligned with reference sequences. PCR was repeated for negative samples, and sequencing repeated for inconclusive results.

### Statistical analysis

Univariate analysis was performed using Chi square test to compare proportions. For comparison of continuous variables, including meteorological data (monthly median rainfall and monthly median temperatures), we used two-sample Wilcoxon rank-sum (Mann-Whitney U) test, since the data did not display a normalized distribution. A P-value ≤ 0.05 was considered statistically significant. Multivariate analysis of characteristic features for infection with *Cryptosporidium* and *G*. *lamblia* included the following nine variables; sex, age, place of residence, parent level of education, duration of diarrhea, hydration status, underweight, stunting and wasting. In multivariate analysis of characteristic features for carriage among controls, we included the same factors except for hydration status and duration of diarrhea. Statistical analysis was performed using Stata 13 (Stata Corp, College Station, TX, USA).

## Results

### Study population

DNA for PCR testing was available for 1259 patients; 701 cases and 558 controls. DNA for PCR testing was insufficient and not available for 4 of the cases and 3 of the controls, and these children were omitted in further analyzes. The distribution of children from each of the study sites was 639 from Ilala, 373 from Kinondoni and 247 from Temeke Municipal district hospital. Of these, 523 were females and 736 were males. The age distribution was as follows: 322 children between 0 to 6 months, 558 children between 7 to 12 months, 248 children between 13 to 18 months, and 131 children between 19 to 24 months. HIV testing results were available for 420 of the children, of whom 33 had a positive test result and 387 had a negative test result.

### Assay performance

The primers and probes have been extensively tested previously [12,20,24], but to assure quality, we tested the assay with known positive samples, for cross-reactivity against other pathogens in the assay, for detection of pathogens in mixed samples, and for efficiency for each target. The multiplex PCR assay resulted in amplification curves for the correct targets as expected. No cross-reactivity was detected, and crosstalk was corrected by applying color compensation.

The efficiency (using the formula E = 10^−1/slope–^ 1) and Error value (E) of the assay for each of the four different targets were: 99.7% and E = 0.013 for *C*. *parvum*/ *hominis*, 97.9% and E = 0.008 for *E*. *histolytica*, 98.5% and E = 0.004 for *G*. *lamblia* and 96.4% and E = 0.018 for PhHV–1.

No amplification of any of the no-template controls was detected.

### Prevalence of the protozoans

The overall prevalence of protozoans in the study population was 14.9% (187/1259), of which 19.7% (138/701) of the cases and 8.8% (49/558) of the controls tested positive for one or two of the three protozoans. The prevalence of *C*. *parvum*/ *hominis* was significantly higher in cases (16.3%, 114/701) than in controls (3.1%, 17/558; P < 0.001; OR = 6.2; 95% CI: 3.7–10.4). The prevalence of *G*. *lamblia* was significantly higher in controls (6.1%, 34/558) than in cases (3.4%, 24/701; P = 0.027; OR = 1.8; 95% CI: 1.1–3.1). Two samples were positive for both *Cryptosporidium* and *G*. *lamblia*, but all samples were negative for *E*. *histolytica*.

### Identification of *Cryptosporidium* species

Species identification of the *C*. *parvum*/ *hominis* positive samples resulted in 10 *C*. *parvum* samples (7.6%) and 111 *C*. *hominis* samples (84.7%). For ten of the samples it was not possible to identify whether they were *C*. *parvum* or *C*. *hominis*.

### Genotyping of *G*. *lamblia*

A PCR product that could be sequenced was only achieved for 13 of the 58 *G*. *lamblia* positive samples. Of these 13 PCR products, only 8 gave sequences that could be analyzed, of which 4 was identified as assemblage A and 4 as assemblage B.

### Characteristics of infection with *Cryptosporidium*

Among study participants with known HIV-status, including both cases and controls, *Cryptosporidium* infection was significantly more prevalent in HIV-positive (24.2%, 8/33) than in HIV-negative (3.9%, 15/387) children in univariate analysis (P < 0.001; OR = 7.9; 95% CI: 3.1–20.5). In multivariate analysis of this same part of the study population, including both HIV- and *Cryptosporidium* infection, HIV-positive status was still significantly associated with *Cryptosporidium* infection (P = 0.001; OR = 5.6; 95% CI: 2.1–15.3), while stunting was not (P = 0.07). All the ten *C*. *parvum* positive samples were from HIV-negative children. The characteristics of cases and controls tested for *C*. *parvum*/ *hominis* are shown in Table 2. When we analyzed cases and controls separately, multivariate analysis of cases showed that stunting was the only characteristic significantly associated with *Cryptosporidium* infection (P = 0.007; OR = 2.12; 95% CI: 1.2–3.8).

**Table 2:** Characteristics of infection with *C*. *parvum*/ *hominis* in children in Dar es Salaam, Tanzania, and results from univariate and multivariate logistic regression.

Characteristics			Cases			Controls		
	N	N		Univariate	Multivariate		Univariate	Multivariate
	Cases	Controls	n (%)	OR (95% CI)	OR (95% CI)	n (%)	OR (95% CI)	OR (95% CI)
**Sex**							
Male	432	304	75 (17.4)	1.24 (0.81–1.89)	1.24 (0.81–1.91)	6 (2.0)	0.44 (0.16–1.22)	0.48 (0.17–1.34)
Female	269	254	39 (14.5)	1	1	11 (4.3)	1	1
**Age**							
< 12 months	537	343	90 (16.8)	1		11 (3.2)	1	1
> 12 months	164	215	24 (14.6)	0.85 (0.52–1.39)	0.76 (0.45–1.28)	6 (2.8)	0.87 (0.32–2.38)	1.15 (0.39–3.37)
**Place of residence**							
Kinondoni	211	162	43 (20.4)	1.25 (0.73–2.14)	1.21 (0.70–2.11)	5 (3.1)	0.72 (0.19–2.74)	0.66 (0.16–2.66)
Ilala	337	302	45 (13.5)	0.75 (0.44–1.27)	0.83 (0.48–1.44)	8 (2.7)	0.61 (0.18–2.08)	0.54 (0.15–1.88)
Temeke	153	94	26 (17.0)	1	1	4 (4.3)	1	1
**Parent level of education**							
Higher level	23	7	3 (13.0)	1	1	1 (14.3)	1	1
Secondary	138	130	22 (15.9)	1.26 (0.35–4.62)	1.38 (0.37–5.13)	5 (3.9)	0.24 (0.02–2.39)	0.22 (0.02–2.41)
Primary and below	540	421	89 (16.5)	1.32 (0.38–4.52)	1.41 (0.40–4.95)	11 (2.6)	0.16 (0.02–1.45)	0.16 (0.02–1.60)
**Type of diarrhea**							
Acute diarrhea	623	NA	95 (15.3)	1	1	NA	NA	NA
Persistent diarrhea	78	NA	19 (24.4)	1.79 (1.02–3.14)*	1.51 (0.84–2.71)	NA	NA	NA
**Hydration status**								
No dehydration	172	NA	32 (18.6)	1	1	NA	NA	NA
Presence of dehydration	529	NA	82 (15.5)	0.80 (0.51–1.26)	0.95 (0.59–1.53)	NA	NA	NA
**Nutritional status**								
***WAZ***								
Normal weight	301	338	42 (14.0)	1	1	13 (3.9)	1	1
Underweight	400	220	72 (18.0)	1.35 (0.89–2.05)	1.10 (0.68–1.77)	4 (1.8)	0.46 (0.15–1.44)	0.55 (0.14–2.12)
***LAZ***							
Normal	223	256	23 (10.3)	1	1	8 (3.1)	1	1
Stunted	478	302	91 (19.0)	2.04 (1.25–3.33)**	2.12 (1.18–3.79)*	9 (3.0)	0.95 (0.36–2.51)	1.26 (0.42–3.84)
***WLZ***								
Normal	491	441	80 (16.3)	1	1	16 (3.6)	1	1
Wasting	210	117	34 (16.2)	0.99 (0.64–1.54)	1.41 (0.81–2.44)	1 (0.9)	0.23 (0.03–1.74)	0.31 (0.04–2.74)

N: Total number of samples tested; n: number of positive samples; NA: not applicable for controls; WAZ: Weight-for age-Z-score; LAZ: Length-for-age-Z-score; WLZ: Weight-for-length-Z-score

*: P < 0.05;

**: P < 0.01

**Fig 1:**
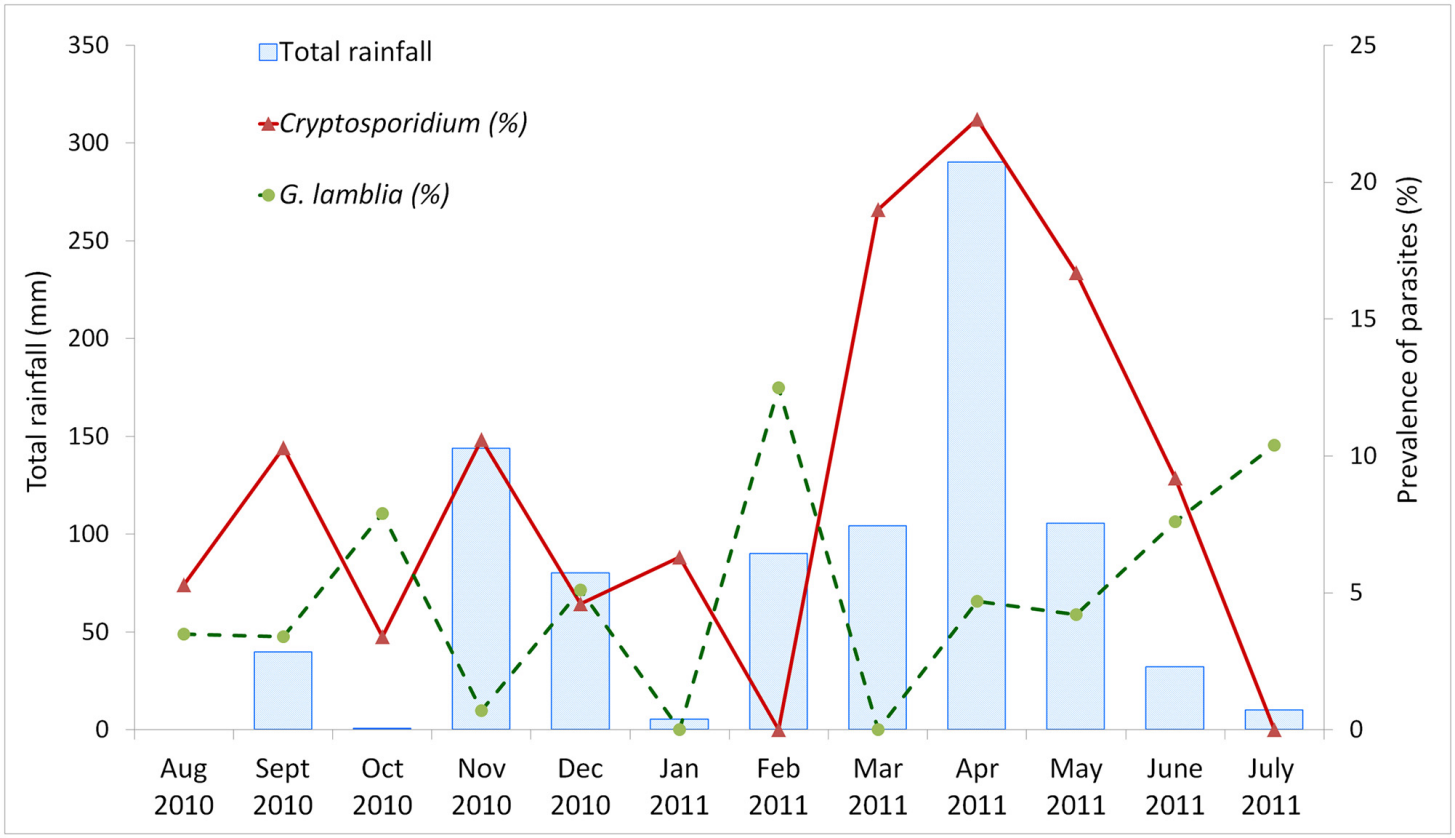
Prevalence of *Cryptosporidium* infection, *G*. *lamblia* infection and total rainfall each month during the study period. The graph shows the prevalence (%) of *Cryptosporidium* infection and *G*. *lamblia* infection among all study participants, together with the total rainfall in mm, for each month during the study period.

**Fig 2:**
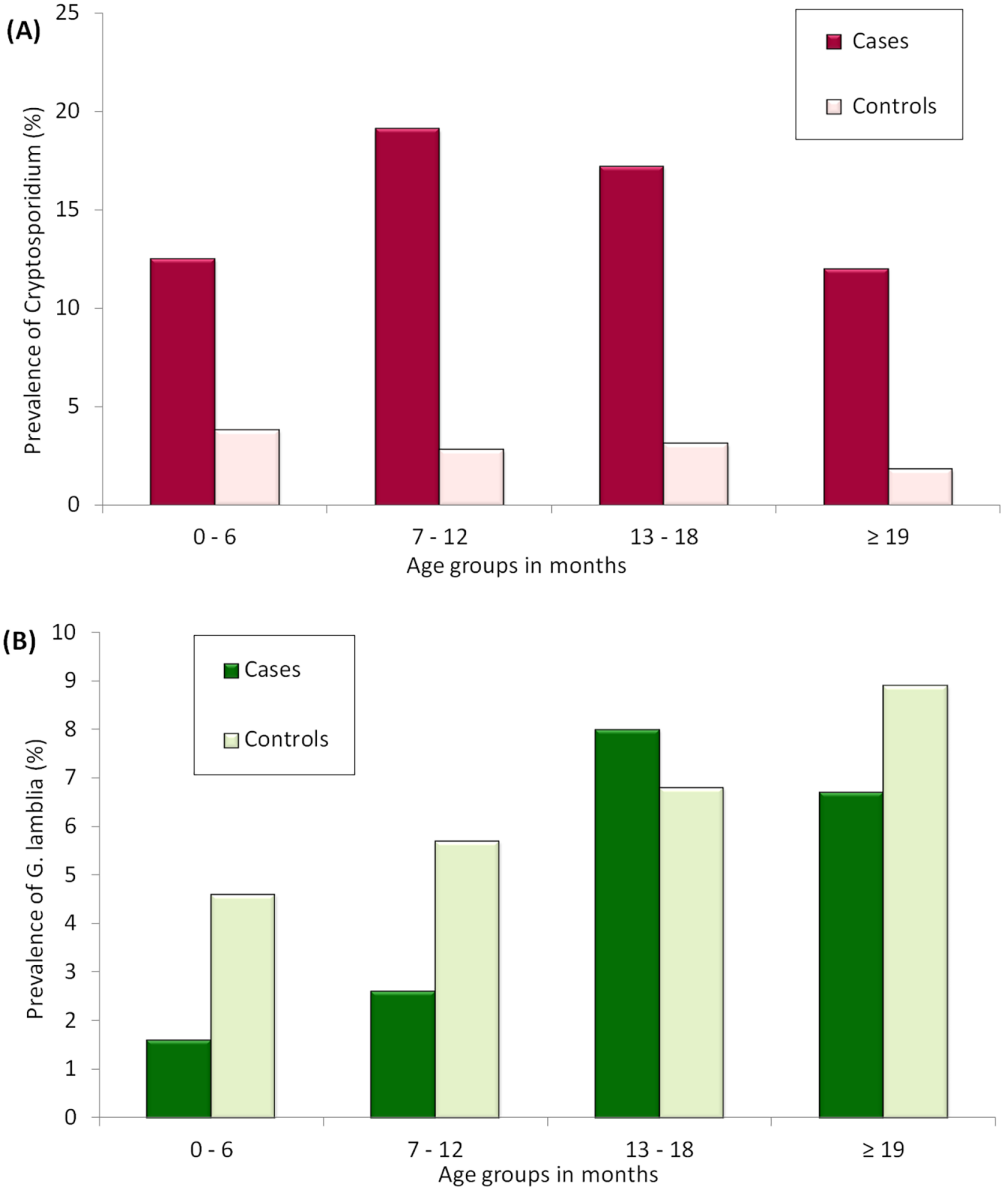
Prevalence of *Cryptosporidium* and *G*. *lamblia* for the different age groups. The graph shows the prevalence of (A) *Cryptosporidium* infection and (B) *G*. *lamblia* infection for the different age groups.

The prevalence of *Cryptosporidium* was higher in the rainy months (12.9%, 105/812, median rainfall 105 mm per month) than in the dry months (5.8%, 26/447, median rainfall 78 mm rainfall per month (P <0.001; Fig 1). There was no significant difference in prevalence of *Cryptosporidium* between the cool months (8.9%, 53/594, median temperature 27°C) and the hot months (11.7%, 78/605, median temperature 27°C; P = 0.92). The median age for both *Cryptosporidium* infected and uninfected children was 10 months (P = 0.399). The prevalence of *Cryptosporidium* in different age groups is illustrated in Fig 2A. Information on breastfeeding was only available for the age group 7–12 months, in which data were collected for 475/ 558 children (both cases and controls). There was no difference in prevalence of *Cryptosporidium* infection between children who were breastfed or those who were not, neither among cases, controls or the total study population.

### Characteristics of infection with *G*. *lamblia*

*G*. *lamblia* infection was more prevalent in the cool months (6.4%, 38/594), than in the hot months (3.0%, 20/665; P = 0.004; OR = 2.2; 95% CI: 1.3–3.8), but with a prevalence of 4.2% (34/812) in rainy season and 5.4% (24/447) in dry season, it was not significantly affected by rainfall (P = 0.338). As seen in Table 3 and Fig 2B, the prevalence of *G*. *lamblia* infection in controls increased with age, and among cases age > 12 months was significantly associated with a higher *G*. *lamblia* prevalence (P = 0.003; OR = 3.5; 95% CI: 1.5–7.8). Among children < 12 months, *G*. *lamblia* infection was more prevalent in controls than in cases, (P = 0.02), but this association was not significant in children > 12 months.

**Table 3:** Characteristics of infection with *G*. *lamblia* in children in Dar es Salaam, Tanzania, and results from univariate and multivariate logistic regression.

Characteristics			Cases			Controls		
	N	N		Univariate	Multivariate		Univariate	Multivariate
	Cases	Controls	n (%)	OR (95% CI)	OR (95% CI)	n (%)	OR (95% CI)	OR (95% CI)
**Sex**								
Male	432	304	18 (4.2)	1.91 (0.75–4.86)	1.65 (0.63–4.37)	15 (4.9)	0.64 (0.32–1.29)	0.60 (0.30–1.23)
Female	269	254	6 (2.2)	1	1	19 (7.5)	1	1
**Age**								
< 12 months	537	343	12 (2.2)	1	1	18 (5.3)	1	1
> 12 months	164	215	12 (7.3)	3.45 (1.52–7.84)**	3.23 (1.35–7.73)**	16 (7.4)	1.45 (0.72–2.91)	1.65 (0.78–3.49)
**Place of residence**								
Kinondoni	211	162	1 (0.5)	0.09 (0.01–0.70)*	0.09 (0.01–0.74)*	10 (6.2)	2.00 (0.54–7.44)	1.97 (0.52–7.50)
Ilala	337	302	15 (4.5)	0.84 (0.35–2.04)	0.79 (0.31–2.00)	21 (7.0)	2.27 (0.66–7.78)	2.13 (0.61–7.41)
Temeke	153	94	8 (5.2)	1	1	3 (3.2)	1	1
**Parent level of education**								
Higher level	23	7	1 (4.4)	1	1	0 (0.0)	ND	ND
Secondary	138	130	4 (2.9)	0.66 (0.07–6.15)	0.49 (0.05–5.10)	3 (2.3)	ND	ND
Primary and below	540	421	19 (3.5)	0.80 (0.10–6.27)	0.52 (0.06–4.63)	31 (7.4)	ND	ND
**Type of diarrhea**								
Acute diarrhea	623	NA	21 (3.4)	1	1	NA	NA	NA
Persistent diarrhea	78	NA	3 (3.9)	1.15 (0.33–3.94)	2.1 (0.54–8.06)	NA	NA	NA
**Hydration status**								
No dehydration	172	NA	3 (1.7)	1	1	NA	NA	NA
Presence of dehydration	529	NA	21 (4.0)	2.33 (0.69–7.9)	2.17 (0.59–7.94)	NA	NA	NA
**Nutritional status**								
*** WAZ***								
Normal weight	301	338	12 (4.0)	1	1	23 (6.8)	1	1
Underweight	400	220	12 (3.0)	0.75 (0.33–1.68)	0.52 (0.18–1.47)	11 (5.0)	0.72 (0.34–1.51)	0.65 (0.25–1.67)
*** LAZ***								
*** ***Normal	223	256	9 (4.0)	1	1	14 (5.5)	1	1
*** ***Stunted	478	302	15 (3.1)	0.77 (0.33–1.79)	1.01 (0.37–2.79)	20 (6.6)	1.22 (0.61–2.48)	1.40 (0.63–3.1)
***WLZ***								
Normal	491	441	14 (2.8)	1	1	29 (6.6)	1	1
Wasting	210	117	10 (4.8)	1.70 (0.75–3.90)	1.85 (0.63–5.47)	5 (4.3)	0.63 (0.24–1.68)	0.75 (0.24–2.29)

N: Total number of samples tested; n: number of positive samples; NA: not applicable for controls; ND: No data due to few observations; WAZ: Weight-for age-Z-score; LAZ: Length-for-age-Z-score; WLZ: Weight-for-length-Z-score

*: P < 0.05;

**: P < 0.01

*G*. *lamblia* infection was significantly less frequent in children on breastfeeding (1.9%, 7/368) than those not on breastfeeding (6.5%, 7/107; P = 0.012). Among the cases in this same age group, breastfeeding was significantly associated with lower prevalence of *G*. *lamblia* infection, 0.4% (1/225), versus 6.0% (3/50; P = 0.003). Among the controls, the prevalence of *G*. *lamblia* infection did not differ significantly among children who were breastfed (4.2%, 6/143) and those not breastfed (7.0%, 4/57; P = 0.409). The median age for children with *G*. *lamblia* infection was higher than for those without (median 12 months vs. 10 months; P = 0.001). Characteristics for cases and controls that had a *G*. *lamblia* infection are shown in Table 3.

## Discussion

Protozoans such as *Cryptosporidium*, *E*. *histolytica* and *G*. *lamblia* are all common causes of diarrheal illness worldwide, particularly in children. In this case-control study we targeted young children in Dar es Salaam, Tanzania, and found overall a quite high prevalence of these intestinal parasites.

The prevalence of *Cryptosporidium* infection is comparable to that found in children in other studies in sub-Saharan Africa, both in the Global Enteric Multicenter Study (GEMS), and in the study of Mbae et al. in Kenya [5,11]. The higher prevalence in cases than in controls concurs with findings from Kenya and supports the notion that the parasite causes symptomatic diarrhea [11]. In contrast, the study by Vargas et al. found that only one out of 451 hospitalized children < 5 years of age in Kilombero district in Tanzania had infection with *Cryptosporidium* [13]. This could be due to differences in methodology, using microscopy which is generally known to be less sensitive, although regional differences may exists.

Cryptosporidiosis is often linked to impaired immunity, particularly HIV-associated immunosuppression, hence many of the studies on *Cryptosporidium* prevalence have been performed on HIV-positive patients, most often including adults, but also children [25]. In our study we found an association between *Cryptosporidium* infection and HIV-status in small children. Although the HIV-status was only known for approximately one third of the children, HIV-positive children were almost eight times more likely to have *Cryptosporidium* than those who were HIV-negative. The study among Kenyan children also found this association, but with an odds ratio of 3.1 [11]. A study of Ugandan children with persistent diarrhea, found that HIV-positive children were 18 times more likely to have *Cryptosporidium* than those who were HIV-negative [26]. However, *Cryptosporidium* should not be ignored as a cause of diarrhea in small children not known to be HIV-positive, as the GEMS-study found that it was an important pathogen at all sites regardless of HIV-prevalence, and the second most common pathogen causing diarrhea in infants [5].

The interaction between diarrhea and malnutrition is complex and multifactorial [2,27]. In univariate analysis, we found persistent diarrhea to be significantly more prevalent among cases infected with *Cryptosporidium*, and this concurs with other studies reporting an association between cryptosporidiosis and prolongation of diarrhea [26,28]. Stunting affects millions of children in developing countries [29]. Persistent diarrhea increases the risk of stunting. Molloy et al. found association between stunting and *Cryptosporidium* infection among Nigerian children [30], and Yones et al. found the same association among Egyptian children [31], which supports our findings that stunted children had significantly higher risk of being infected with *Cryptosporidium*. However, any causal relationship between stunting and *Cryptosporidium* infection could not be established in the current study. Indeed, when analyzing the part of the study population with known HIV-status, cryptosporidiosis was only associated with HIV-positive status, and not with stunting. This should however be interpreted with caution, as the HIV-status was known for only a limited number of the children infected with *Cryptosporidium*.

Species identification of the isolates showed that the majority were *C*. *hominis*, while only 7.6% were *C*. *parvum*. Ten remaining isolates could not be identified to the species level, likely due to low amount of target. The prevalence of *C*. *hominis* and *C*. *parvum* varies in different parts of the world [25], and risk factors are also reported to differ [25,30,32]. A predominance of *C*. *hominis*, with a ratio between *C*. *hominis* and *C*. *parvum* not much different from what we report, has also been reported from pediatric populations in other developing countries and *C*. *hominis* seems to be the dominating species in sub-Saharan Africa [26,33–36]. Our finding of *C*. *hominis* predominating contrasts a previous study from the same area, where only *C*. *parvum* was reported, with a prevalence of 18.9% in children < 5 years of age [14]. In that study a rapid test was used for detecting *Cryptosporidium*, for which the manufacturer claims it detects *C*. *parvum*. However, this rapid test also detects *C*. *hominis*, thus some *C*. *parvum* isolates in that study may have been misclassified isolates of *C*. *hominis* [37, 38]. The probe we used in the multiplex-PCR only target *C*. *hominis* and *C*. *parvum*, hence other species, like *C*. *meleagridis*, *C*. *felis* and *C*. *canis*, which are also known to cause infection in humans [39], could not be detected.

*Cryptosporidium* infection was significantly more prevalent in the rainy season than in the dry season, and this is supported by previous studies [11,18,40].

The majority of *Cryptosporidium* positive samples were from children younger than one year of age, but age was not a significant factor. This might not be very surprising since all the study participants were below 2 years of age, and other studies which also included older children have reported a higher prevalence of infection among younger children, those below 2 years of age in particular [5,11,34].

The prevalence of *G*. *lamblia* in different regions, including sub-Saharan Africa, shows large differences [11–13,15,18,31]. The prevalence in our study population (4.6%) was higher than reported in another study from the same area (1.9%) [14]. However, considering the different study population (children with diarrhea only), and use of a less sensitive detection method (rapid test), the prevalence may not be significantly different from that of 3.4% found among the cases in our study. Several studies have reported that *G*. *lamblia* infection shows seasonality, with a higher prevalence during the rainy season [13,18,41]. We did not find this association with rainfall in our study. In contrast, we found a significantly higher prevalence of *G*. *lamblia* infection in the cool season than in the hot season, though with a low odds ratio. In a study of children in Thailand, Wongstitwilairoong et al. reports a higher prevalence of intestinal parasites in the cool season than in the hot season, however the authors did not provide data for *G*. *lamblia* alone [42]. Since most studies relate seasonality with rainfall and not temperature, there is some uncertainty whether any effect of temperature was considered.

The prevalence of *G*. *lamblia* was significantly higher in controls than in cases. This is in agreement with other studies in pediatric populations [5,11,15,43]. The controls did not have diarrhea for the last month prior to enrollment, and hence were asymptomatic carriers. In our study we did not see any statistical difference in nutritional status between infected cases and controls, although the prevalence of underweight and stunting was slightly higher among cases, while the prevalence of wasting was almost equal.

Several studies report that the prevalence of *G*. *lamblia* infection in children increases with age [11,12,43]. Although our study included children within a limited age range, age > 1 year was significantly associated with a higher prevalence among cases, and the prevalence also appeared to increase with age among controls, though this relationship was not statistically significant.

Information on breastfeeding was available for the majority of the children aged 7–12 months. With a significantly lower prevalence of *G*. *lamblia* infection among those on breastfeeding, as well as a significantly lower prevalence among the cases in this age group, breastfeeding seems to protect children with diarrhea from symptomatic *G*. *lamblia* infection. The fraction of *G*. *lamblia* infection was higher among controls regardless of being breastfed or not, suggesting that breastfeeding does not prevent asymptomatic infection. However, Mahmud et al. found that breastfeeding prevents both symptomatic and asymptomatic *G*. *lamblia* infection among children < 1 year in Egypt [44]. Ignatus et al. also found a protective effect of breast-milk in a study of Rwandan children, but did not relate to symptomatic or asymptomatic infection [12].

Identification of the isolates as assemblages A or B using the TPI gene were, after several attempts, unfortunately only achievable for 8 of the 58 isolates, showing equal prevalence of these two assemblages. With this low outcome, conclusions related to type cannot be drawn. The multiplex Real-Time PCR, targeting a multicopy gene, showed high Cq-values for the majority of isolates. The TPI gene used for typing is a single copy gene, and this could contribute to the low sensitivity. Other explanation could be sequence variability, leading to primer mismatches.

In this study population we did not find any *E*. *histolytica*. Studies from sub-Saharan countries have reported different prevalence of *E*. *histolytica* [11,13,45,46]. However, several of these studies may overestimate the prevalence as they depended on microscopy, which cannot distinguish between *E*. *histolytica* and the non-pathogenic *E*. *dispar*. A study from the Kilimanjaro district, Tanzania, reported an *E*. *histolytica* prevalence of 0.8% [45]. Prevalence of *E*. *histolytica*/*dispar* has been reported to increase with age [11,45], and this agrees with the low prevalence found in young children in our study, as well as other studies, like the study by Nesbitt et al., and the probably very low prevalence found by Vargas et al.. The GEMS-study did not report on any *E*. *histolytica* among children < 2 years of age from the sub-Saharan African countries, neither did Krumkamp et al. in a study of children 0–13 years from Ghana, which also supports our results [5,43].

This is the first study from Tanzania reporting on the prevalence of protozoans in a large study population of children < 2 years of age, with and without diarrhea, and not many exist from other sub-Saharan countries. The seemingly protective effect of *G*. *lamblia* in healthy children needs further elucidation, as it could give important knowledge into the potential immunomodulatory host-pathogen interaction of this microbe. Further studies should also include other causes of diarrhea, such as other parasites, bacteria and viruses, including even larger study groups and older children, in order to broaden our understanding of childhood diarrhea.

## Supporting Information

S1 Checklist STROBE checklist. (DOC)
