# Supplementary material for: Prevalence of Cryptosporidium parvum/hominis, Entamoeba histolytica and Giardia lamblia among Young Children with and without Diarrhea in Dar es Salaam, Tanzania
Source: PLoS Negl Trop Dis. 2015 Oct 9;9(10):e0004125. doi: 10.1371/journal.pntd.0004125 (PMC4599730; doi:10.1371/journal.pntd.0004125)
Supplement: S1 Checklist — (DOC) [file pntd.0004125.s001.doc]

STROBE Statement—Checklist of items that should be included in reports of ***case-control studies***

|  | Item No | Recommendation |
| --- | --- | --- |
| **Title and abstract** | 1 | (*a*) Indicate the study’s design with a commonly used term in the title or the abstract  **Line no. 36: We performed an unmatched case-control study…….** |
|  |  | (*b*) Provide in the abstract an informative and balanced summary of what was done and what was found  **Line no. 36- …..(Methodology/Principal Findings)** |
| Introduction | | |
| Background/rationale | 2 | Explain the scientific background and rationale for the investigation being reported  **Line no. 32 (Abstract; Background)**  **See Introduction**  **Line no. 102 - ……** |
| Objectives | 3 | State specific objectives, including any prespecified hypotheses  **Line no. 108: The objectives of the present study were to investigate the prevalence of *C*. *parvum*/ *hominis*, *E.* *histolytica* and *G. lamblia* among young children in Dar es Salaam, Tanzania, and to identify risk factors for infection.** |
| Methods | | |
| Study design | 4 | Present key elements of study design early in the paper  **Line no. 36: We performed an unmatched case-control study among children < 2 years of age in Dar es Salaam, recruited from August 2010 to July 2011.**  **Line no. 122: The study population and data collection have previously been described [19]. Briefly, this prospective study was performed between August 2010 and July 2011, in Dar es Salaam, Tanzania, covering both the dry and the wet seasons. A total of 1266 children < 2 years of age were recruited.**  **Line no. 128: Cases (N = 705) were children admitted due to diarrhea at one of the three major hospitals in Dar es Salaam; Muhimbili National Hospital, Amana and Temeke Municipal district hospitals. Controls (N = 561) were children with no history of diarrhea during the last month prior to enrollment.** |
| Setting | 5 | Describe the setting, locations, and relevant dates, including periods of recruitment, exposure, follow-up, and data collection  **Line no. 122: The study population and data collection have previously been described [19]. Briefly, this prospective study was performed between August 2010 and July 2011, in Dar es Salaam, Tanzania, covering both the dry and the wet seasons.**  **Line no. 128:** **Cases (N = 705) were children admitted due to diarrhea at one of the three major hospitals in Dar es Salaam; Muhimbili National Hospital, Amana and Temeke Municipal district hospitals.** |
| Participants | 6 | (*a*) Give the eligibility criteria, and the sources and methods of case ascertainment and control selection. Give the rationale for the choice of cases and controls  **Line no. 124: A total of 1266 children < 2 years of age were recruited. Diarrhea was defined as three or more watery stools within 24 hours. An episode of diarrhea was considered over when two consecutive days pass without diarrhea. An episode of acute diarrhea was defined as duration between 24 hours and less than 14 days. Persistent diarrhea was defined as diarrhea for 14 days or more.**  **Line no. 130:** **Controls (N = 561) were children with no history of diarrhea during the last month prior to enrollment.**  **The case definition is commonly used in many studies of this kind.**  **Duration of one month without diarrhea for controls might seem long in this setting, but this duration was chosen to ascertain the controls were free from carrying any pathogens of previous diarrheal episodes.** |
|  |  | (*b*) For matched studies, give matching criteria and the number of controls per case  **Not applicable** |
| Variables | 7 | Clearly define all outcomes, exposures, predictors, potential confounders, and effect modifiers. Give diagnostic criteria, if applicable  **Main outcomes were prevalence of *C*. *parvum*/ *hominis*, *E.* *histolytica* and *G. lamblia*, as diagnosed by PCR.**  **A positive test for any of these pathogens defined the exposure to the clinical data obtained, including: age, gender, place of residence, parent level of education, type of diarrhea, hydration status and nutritional status. Effect modification of seasonality and the subgroup with HIV status was examined. Outcomes, exposures, and effect modifiers are defined in Methods from line 121 onwards and in Table 2.** |
| Data sources/ measurement | 8* | For each variable of interest, give sources of data and details of methods of assessment (measurement). Describe comparability of assessment methods if there is more than one group  **Line 131. Clinical data were collected by means of a common questionnaire filled in by health personnel upon inclusion of the participants. For cases data were also collected from patient files. For diagnosis of parasitic infection see**  **Line no. 146: see “Sample material”**  **Line no. 150: see “Multiplex real-time PCR for detection of protozoans”**  **Line no. 178: see “Identification of Cryptosporidium species”**  **Line no. 194: see “Genotyping of Giardia”** |
| Bias | 9 | Describe any efforts to address potential sources of bias  **All consecutive, eligible children were asked to participate in the study. Lab personnel were blinded for any clinical data.**  **Line no. 170: Each run included….**  **Line no. 173: The PCR was repeated for samples with weak positive….**  **Line no. 174: A unidirectional workflow pre- to post-PCR…..**  **Line no. 189: Each run included….**  **Line no. 190: For samples that were negative….**  **Line no. 205: PCR was repeated….**  **Line no. 233: see “Assay performance”** |
| Study size | 10 | Explain how the study size was arrived at  **The paper is part of a larger study of multiple diarrheagenic organisms. Sample size was estimated with respect to being able to produce a point estimate for prevalence with 5% uncertainty for a number of pathogens, as well as to detect differences among cases and controls and among cases in hot versus cold seasons and rainy versus dry seasons. The expected prevalence of various etiologies may vary, and a number of different scenarios were calculated. For the purpose of simplifying sample-size calculation we assumed the published prevalence of 18.5% for Rotavirus as a reference (ref Moyo et al 2007) and calculated the following sample sizes using the “power” command in Stata (Stata Corp, College Station, TX, USA):**  **One-sample calculation with estimate of prevalence, anticipated prevalence of 18.5%, precision 5 percent points in each direction (18.5% vs 13.5% and 18.5% vs 23.5%), power 80% resulted in a sample size of n=470 (500+440/2)**  **Two-sample calculation to detect a difference of 10 percent points between groups defined by season / rainfall, with a power of 80%, resulted in a sample size of 472 cases (236 in each group).**  **Considering multiple comparisons of multiple etiologies with different prevalences, we increased the sample size arbitrarily by 200, arriving at 670 which was perceived as a feasible sample size. To allow for similarly calculations among healthy controls, we decided to include cases and healthy controls at a 1:1 ratio, arriving at a total sample size of 1340, half cases and half controls.** |
| Quantitative variables | 11 | Explain how quantitative variables were handled in the analyses. If applicable, describe which groupings were chosen and why  **Handling of quantitative variables including temperatures, rainfall and age are described from line 209 onwards under the heading Statistical analysis.** |
| Statistical methods | 12 | (*a*) Describe all statistical methods, including those used to control for confounding  **Line no. 208: see Statistical analysis** |
|  |  | (*b*) Describe any methods used to examine subgroups and interactions  **Line no. 208: see Statistical analysis** |
|  |  | (*c*) Explain how missing data were addressed  **Line no. 223: DNA for PCR testing was insufficient and not available for 4 of the cases and 3 of the controls, and these children were omitted in further analyzes.** |
|  |  | (*d*) If applicable, explain how matching of cases and controls was addressed  **Not applicable** |
|  |  | (*e*) Describe any sensitivity analyses  **Not applicable** |
| Results | | |
| Participants | 13* | (a) Report numbers of individuals at each stage of study—eg numbers potentially eligible, examined for eligibility, confirmed eligible, included in the study, completing follow-up, and analysed  **Line no. 223: DNA for PCR testing was available for 1259 patients; 701 cases and 558 controls. DNA for PCR testing was insufficient and not available for 4 of the cases and 3 of the controls, and these children were omitted in further analyzes.** |
|  |  | (b) Give reasons for non-participation at each stage  **Not applicable** |
|  |  | (c) Consider use of a flow diagram  **Not applicable** |
| Descriptive data | 14* | (a) Give characteristics of study participants (eg demographic, clinical, social) and information on exposures and potential confounders  **Line no. 222: see “Study population”**  **See Table 2. Characteristics of infection with *C. parvum*/ *hominis* in children in Dar es Salaam, Tanzania, and results from univariate and multivariate logistic regression.**  **See Table 3. Characteristics of infection with *G. lamblia* in children in Dar es Salaam, Tanzania, and results from univariate and multivariate logistic regression.** |
|  |  | (b) Indicate number of participants with missing data for each variable of interest  **Line no. 229: HIV testing results….**  **Line no. 283: Information on breastfeeding was only available….**  **See Table 2. Characteristics of infection with *C. parvum*/ *hominis* in children in Dar es Salaam, Tanzania, and results from univariate and multivariate logistic regression.**  **See Table 3. Characteristics of infection with *G. lamblia* in children in Dar es Salaam, Tanzania, and results from univariate and multivariate logistic regression.** |
| Outcome data | 15* | Report numbers in each exposure category, or summary measures of exposure  **Line no. 246: see “Prevalence of the protozoans”** |
| Main results | 16 | (*a*) Give unadjusted estimates and, if applicable, confounder-adjusted estimates and their precision (eg, 95% confidence interval). Make clear which confounders were adjusted for and why they were included  **Line no. 246: see “Prevalence of the protozoans”**  **Line no. 265: see ” Characteristics of infection with *Cryptosporidium***  **Line no. 296: Characteristics of infection with *G. lamblia*** |
|  |  | (*b*) Report category boundaries when continuous variables were categorized  **Table 2 and Table 3. The continuous variable age was categorised into <1 year and >1 year of age.**  **Line 133-136: Weight for age (WAZ), length for age (LAZ) and weight for length (WLZ) Z-scores were calculated using EPI Info. Children were categorized to have normal nutritional status, mild or severe malnutrition using Z-scores according to WHO criteria** |
|  |  | (*c*) If relevant, consider translating estimates of relative risk into absolute risk for a meaningful time period  **Not applicable** |

| Other analyses | | 17 | Report other analyses done—eg analyses of subgroups and interactions, and sensitivity analyses  **The same associations as were done for the whole sample set were also done for the subgroup of HIV-positive, and is reported in the manuscript from line no. 266.** |  | |
| --- | --- | --- | --- | --- | --- |
| Discussion | | | | |  |
| Key results | 18 | | Summarise key results with reference to study objectives  **Line no. 317: In this case-control study we targeted young children in Dar es Salaam, Tanzania, and found overall a quite high prevalence of these intestinal parasites.** | | |
| Limitations | 19 | | Discuss limitations of the study, taking into account sources of potential bias or imprecision. Discuss both direction and magnitude of any potential bias  **Line no. 350: However, any causal relationship between stunting and *Cryptosporidium* infection could not be established in the current study. Indeed, when analyzing the part of the study population….**  **Line no. 412: Identification of the isolates as assemblages A or B using the TPI gene were, after several attempts, unfortunately only achievable for 8 of the 58 isolates, showing equal prevalence of these two assemblages. With this low outcome, conclusions….** | | |
| Interpretation | 20 | | Give a cautious overall interpretation of results considering objectives, limitations, multiplicity of analyses, results from similar studies, and other relevant evidence  **Line no. 315: see “Discussion”; The pathogens are discussed with reference to the different risk factors and variables, and with reference to literature.** | | |
| Generalisability | 21 | | Discuss the generalisability (external validity) of the study results  **Line no. 430: This is the first study from Tanzania reporting on the prevalence of protozoans in a large study population of children < 2 years of age, with and without diarrhea, and not many exist from other sub-Saharan countries.**  **Our findings reflect data from a number of other studies in Africa and South-East Asia. Clearly there might be geographical and seasonal variation in various regions, but with the considerable sample size the results are likely to be generalizable to young children hospitalized with diarrhea in Tanzania, and possibly in the rest of eastern Africa.** | | |
| Other information | | | | |  |
| Funding | 22 | | Give the source of funding and the role of the funders for the present study and, if applicable, for the original study on which the present article is based  **This study was supported by the University of Bergen, Norway, and by the National Centre for Tropical Infectious Diseases, Haukeland University Hospital, Norway. The funders had no role in study design, data collection and analysis, decision to publish, or preparation of the manuscript.** | |  |

*Give information separately for cases and controls.

**Note:** An Explanation and Elaboration article discusses each checklist item and gives methodological background and published examples of transparent reporting. The STROBE checklist is best used in conjunction with this article (freely available on the Web sites of PLoS Medicine at http://www.plosmedicine.org/, Annals of Internal Medicine at http://www.annals.org/, and Epidemiology at http://www.epidem.com/). Information on the STROBE Initiative is available at http://www.strobe-statement.org.
